# Canine pillar bone bearing traction method of skull external fixator combined with nickel-titanium memory alloy spring for unilateral cleft lip and palate with severe facial skeletal hypoplasia

**DOI:** 10.4314/ahs.v24i3.38

**Published:** 2024-09

**Authors:** Xin Wang

**Affiliations:** Department of Oral, Cangzhou Medical College, Cangzhou, China

**Keywords:** External fixator of skull, nickel titanium memory alloy spring, bone bearing traction of canine pillar, unilateral cleft lip and palate, severe midfacial skeletal dysplasia, clinical research

## Abstract

**Background:**

For unilateral cleft lip and palate severe midfacial skeletal dysplasia, external skull fixator combined with nickel titanium memory alloy spring was used to treat canine pillar bone bearing traction.

**Methodology:**

Patients (n = 200) with unilateral cleft lip and palate and severe midfacial skeletal hypoplasia who were treated in our hospital within three years were divided into control group (treated with trans suture traction osteogenesis) while the observation group was treated with skull external fixator combined with nickel titanium memory alloy spring supporting force traction of canine bone.

**Results:**

After applying significantly different traction forces to the maxillary tissues on both sides of the patient, the patient was treated with appropriate traction correction, orthodontic treatment and moderate traction on the facial arch to effectively reduce the recurrence of the disease after surgery. Compared with the control group, the curative effect in all aspects was better and the asymmetry rate was lower.

**Conclusion:**

Canine strut bone traction method of external skull fixation combined with nickel-titanium memory alloy spring was applied for severe facial skeletal dysplasia caused by unilateral cleft lip and palate. For this purpose, reasonable and scientific treatment methods were provided for early correction and treatment of the disease.

## Introduction

Cleft lip and palate, as a congenital malformation with a relatively high incidence in oral and maxillofacial position, usually causes a certain degree of involvement of soft or hard tissues of the body. There are significant differences in the characteristics of deformity in each growth stage, for which a more standardized and scientific sequence will generally be carried out in time for the treatment of this disease 1. Because congenital factors (such as ideal growth and development trend, etc.) and acquired factors (such as the effect of surgical treatment, etc.) may affect about a quarter of patients with cleft lip and palate disease in the adolescent development stage may have adverse conditions of midfacial bone hypoplasia 2. The clinical symptoms of this condition are mainly depression in the middle of the face and relatively narrow maxillary arch.

At present, the clinical treatment of unilateral cleft lip and palate severe midface skeletal hypoplasia in the disease is mainly treated with osteotomy traction osteogenesis, through the use of external traction device to promote the body's maxillary tissue suitable for forward movement, on this basis, the bone block movement mode is correspondingly improved and optimized, and finally achieve the purpose of improving the coordination and symmetry between the patient's facial tissues of clinical treatment. However, this treatment will cause significant traumatic effects on patients after surgical treatment, but also have a certain degree of interference effect on the potential of their body's bone growth and development. Patients with unilateral cleft lip and palate disease also present with two bone segments on the non-cleft side (interconnected with the nasal septum) as well as on the cleft side (absent connection with the nasal septum) because of the severity of bone defects at the cleft position and the discontinuous arch. Some scholars such as Thierens 3 treated cleft lip and palate by using the bearing point to perform bone bearing capacity facial traction measures in the central plane of midface bone resistance, and the midface bone tissue of cleft lip and palate has a significant forward movement effect. Although the clinical attention to the symptoms of unilateral cleft lip and palate asymmetric deformity is relatively high, and the research data are rich, the research data on carrying out corrective treatment for both maxillary segments are relatively scarce 4. In the course of clinical treatment of unilateral cleft lip and palate severe midfacial skeletal dysplasia, external skull fixator combined with nickel titanium memory alloy spring was used to treat canine pillar bone bearing traction

## Patients and methods

### Patients

Patients (n=200) with unilateral cleft lip and palate with severe mid-facial skeletal dysplasia treated in our hospital over a three-year period (mainly from April 2019 to April 2022) were selected by the random number table method and were not statistically significantly different from each other in terms of basic information (age and gender, etc.) (P>0.05) (Table 1). This study was approved by the ethics committee of Cangzhou Medical College. Signed written informed consents were obtained from all participants before the study. The research plan in this paper is in line with the Helsinki Declaration.

**Table 1:** Basic information

	Male	Female	Age	Age Mean	Postoperative left complete cleft lip and palate	Postoperative right complete cleft lip and palate
Control group	53 cases	47 cases	8-17 years	12.64 ± 2.38 years	59 cases	41 cases
Observation group	51 cases	49 cases	9-16 years	11.37 ± 2.49 years	56 cases	44 cases

### Inclusion criteria involved as follows

(1) The subjects in this study all had non-comprehensive unilateral cleft lip and palate^3^, with clinical symptoms mainly in the form of significant depressions in the middle of the face, mainly in the nose, lower orbits, zygomatic bone and maxilla, or in the form of bony Class III malocclusion or relatively narrow maxillary dental arches.; (2) The cleft lip and palate and other related restorative surgical treatment had ended, and the surgical treatment procedures were carried out by the same clinician; (3) The patient's clinical relevant data (such as traction treatment data) and records were relatively complete; (4) The patient's head three-dimensional CT imaging examination data and data before and after traction treatment were relatively perfect; (4) The patient's follow-up time was less than one year; (6) The patient and his/her family were informed of the relevant contents of this study (such as treatment links, traction operations, etc.), and voluntarily signed a statement agreement with our hospital.

### Exclusion criteria were set as follows

(1) Patients with a history of other maxillofacial related surgical treatment; (2) patients with relatively low compliance and cooperation with follow-up work; (3) Patients with a series of complications occurred during the traction treatment phase.

## Methods

**Figure 1:**
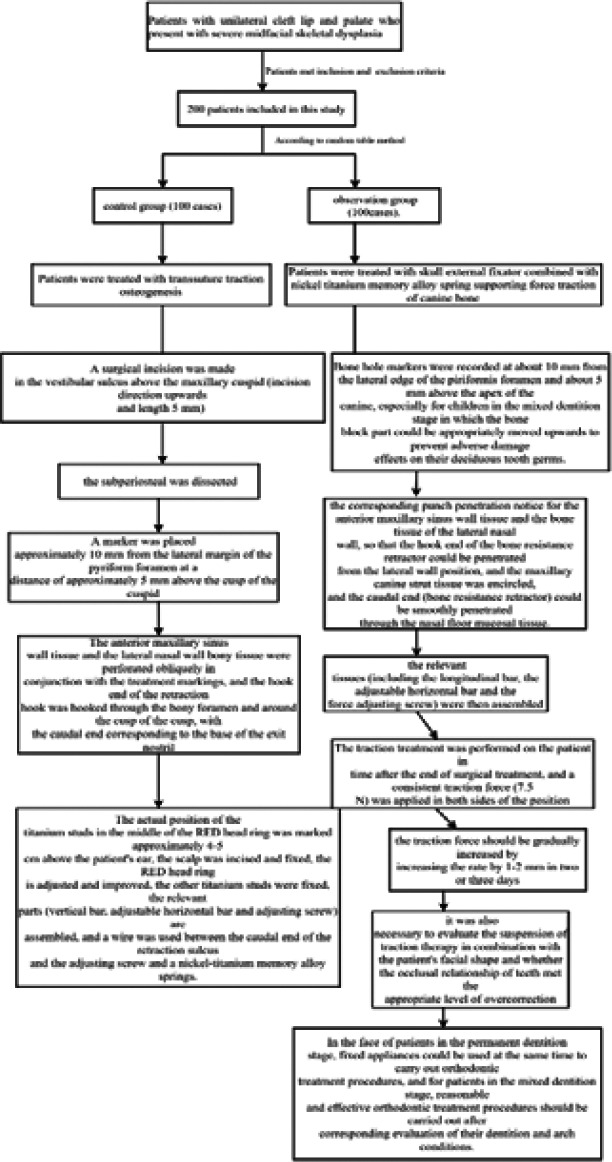
The treatment methods of the study

**In Control group:** Prior to the start of the hand treatment, the patient's imaging data and information were detailed to further clarify the status of the patient's relevant indicators (presence of jaw lesions, actual position of the tooth germ and areas of cranial weakness, etc.). The patient was treated with general anesthesia via oral tracheal intubation and the treatment area was disinfected on the face (75% alcohol) and on the head (0.2% iodophor). Lidocaine at a concentration of 0.5% was applied for bilateral infraorbital foramen blocks and infiltration of the surgical treatment area. A surgical incision was made in the vestibular sulcus above the maxillary cuspid (incision direction upwards and length 5 mm) and the subperiosteal was dissected to ensure that the bilateral pear-shaped foramen margins were exposed to the treatment field. A marker was placed approximately 10 mm from the lateral margin of the pyriform foramen at a distance of approximately 5 mm above the cusp of the cuspid. The anterior maxillary sinus wall tissue and the lateral nasal wall bony tissue were perforated obliquely in conjunction with the treatment markings, and the hook end of the retraction hook was hooked through the bony foramen and around the cusp of the cusp, with the caudal end corresponding to the base of the exit nostril. The actual position of the titanium studs in the middle of the RED head ring was marked approximately 4-5 cm above the patient's ear, the scalp was incised and fixed, the RED head ring is adjusted and improved, the other titanium studs were fixed, the relevant parts (vertical bar, adjustable horizontal bar and adjusting screw) are assembled, and a wire was used between the caudal end of the retraction sulcus and the adjusting screw and a nickel-titanium memory alloy springs(Figure 1).

**In observation group:** (1) Traction device: This product is mainly composed of extracranial stent tractor, nickel-titanium memory alloy spring [made of nickel-titanium memory alloy wire material (with diameter controlled in 1.8 mm); the early length shall be controlled in 23 mm; according to the deformation condition, if the extension length was 1 mm, there would be a certain degree of traction force (about 2.5N)] and bone bearing tractor [made of nickel-titanium alloy (with diameter controlled in 1.56 mm)].

(2) **Surgical treatment procedures:** General anesthesia was performed by endotracheal intubation through oral tissues in all subjects observed in this study, the location of the maxillary vestibular groove was selected to establish a surgical treatment incision, and the subperiosteal stripping arch was carried out to promote the piriformis margin of the body and the anterior wall of the maxillary sinus tissue to be fully exposed under the surgical treatment field. Bone hole markers were recorded at about 10 mm from the lateral edge of the piriformis foramen and about 5 mm above the apex of the canine, especially for children in the mixed dentition stage in which the bone block part could be appropriately moved upwards to prevent adverse damage effects on their deciduous tooth germs. According to the mark points made during the treatment, we carried out the corresponding punch penetration notice for the anterior maxillary sinus wall tissue and the bone tissue of the lateral nasal wall, so that the hook end of the bone resistance retractor could be penetrated from the lateral wall position, and the maxillary canine strut tissue was encircled, and the caudal end (bone resistance retractor) could be smoothly penetrated through the nasal floor mucosal tissue. The extracranial brace retractor was prevented on the patient's head and the head ring was fixed with titanium spikes (approximately 4-5), and the relevant tissues (including the longitudinal bar, the adjustable horizontal bar and the force adjusting screw) were then assembled and the end of the retractor hook and the force adjusting screw were effectively linked together using steel wire and a nickel-titanium memory alloy spring.. Of interest, the distance between the longitudinal bar of the extracranial retractor support and the nasal base should be controlled above 12 cm to ensure that the midface skeleton had sufficient space for forward movement without adversely affecting the patient's view. In addition, the adjustable horizontal bar position should ensure that the early traction mode was mainly between 20 and 30 degrees anteriorly and inferiorly.

(3) **Traction therapy operation:** The traction treatment was performed on the patient in time after the end of surgical treatment, and a consistent traction force (7.5 N) was applied in both sides of the position. After a three-day to five-day adaptation period, the traction force should be gradually increased by increasing the rate by 1-2 mm in two or three days, which should be corresponding to the actual adaptation status and age of the patient. When the patient's midface bone tissue was clearly observed to gradually move anteriorly, it should be combined with the relevant conditions such as bilateral midface depression and forward movement to correspondingly improve the traction force and direction, and finally achieved bilateral independence and asymmetric clinical traction treatment. Secondly, it was also necessary to evaluate the suspension of traction therapy in combination with the patient's facial shape and whether the occlusal relationship of teeth met the appropriate level of overcorrection, while reducing the traction force according to the actual situation and maintaining a certain traction therapy (the maintenance time was usually controlled at 1-3 m) for observation.

After relieving the traction device, for the patients with perfect traction treatment but stable occlusal relationship of teeth, the maintenance treatment was performed again corresponding to this love arch combined with palatal support elastic traction (Figure 1).

**Orthodontic treatment procedures:** The cooperative relationship with orthodontic treatment should always be strengthened during the treatment. If the patient's maxillary advancement was smooth and accompanied by unreasonable arch width (i.e., too wide or too narrow), the palatal support spiral reamer could be used in combination to improve the treatment. In the face of patients in the permanent dentition stage, fixed appliances could be used at the same time to carry out orthodontic treatment procedures, and for patients in the mixed dentition stage, reasonable and effective orthodontic treatment procedures should be carried out after corresponding evaluation of their dentition and arch conditions.

### Evaluation criteria

(1) Three-dimensional CT imaging of the head was performed on the patients before traction therapy (T0) and after the traction transfer was successfully removed (T1), and the corresponding three-dimensional measurement reference plane imaging images were established and improved by selecting the corresponding bone marker position. There were mainly sella imaging center marker points (considered as sphenoid point, S), superior alveolar seat points (A), lowest point of the naso-mandibular suture (INM), lateral margin point of the piriformis foramen (LPA) and pterygomaxillary suture points (PTM). (2) The three-dimensional measurement reference plane mainly included frame ear (FH), coronal plane (CR) and MSR triplane content. (3) Measurement related indicators: the distance between INM, LP and APTM three points and CR plane between the slit side and the non-slit side; the asymmetry rate between INM, LP and APTM.

### Statistical analysis

Statistical Product and Service Solutions (SPSS) 23.0 (IBM, Armonk, NY, USA) was applied for statistical analysis. Independent sample t-test was used for comparison between groups for measurement data obeying normal distribution, and independent sample t-test was used for comparison within groups, all expressed as (x̅±s). Count data were tested by χ^2^ and expressed as rate (%), P<0.05 indicates statistical difference.

## Results

### Clinical treatment effects

Compared with the efficacy of non-slit side before and after traction treatment, the distance between the three points of inferior most point of the nasal-maxillary suture (INM) on the cleft side, lateral edge point of piriformis (LPA) and pterygomaxillary suture point (PTM) on the slit side and CR plane was smaller in the two groups (P < 0.05), but there was no statistical difference in the inferior most point of the nasal-maxillary suture ( INM ) on the cleft side on both sides after treatment (P > 0.05), and compared with the control group, the efficacy in all aspects was better observed (P < 0.05), as shown in Table 2.

**Table 2:** Clinical treatment effects (x̅±s, mm)

	Comparison item	Control group			Observation group		

		INM	LPA	PTM	INM	LPA	PTM
T0	Non-Slit Side	61.02 ± 3.31	56.21 ± 3.44	17.77 ± 3.37	61.23 ± 3.24	57.03 ± 2.69	17.61 ± 3.49
	Slit side	60.05 ± 3.77	51.82 ± 3.99	16.32 ± 4.02	60.46 ± 3.84	53.16 ± 4.05	17.06 ± 3.46
	t	3.496	9.492	2.775	3.416	5.618	3.648
	P	< 0.001	< 0.001	< 0.001	< 0.001	< 0.001	< 0.001
T1	Non-Slit Side	70.21 ± 4.75	65.08 ± 5.78	26.21 ± 6.02	69.64 ± 2.64	65.34 ± 5.34	25.06 ± 5.13
	Slit side	70.17 ± 4.82	63.23 ± 5.59	26.38 ± 5.65	69.08 ± 2.46	64.26 ± 3.49	24.99 ± 4.31
	t	0.276	6.713	- 0.205	0.648	5.315	0.364
	P	0.985	< 0.001	0.961	0.946	< 0.001	0.587
Difference between non-slit side and slit side	T0	0.89 ± 1.54	4.41 ± 2.45	1.56 ± 2.83	0.86 ± 1.42	4.31 ± 2.16	1.26 ± 2.64
	T1	0.03 ± 1.17	2.89 ± 2.22	- 0.08 ± 1.83	0.46 ± 1.62	3.64 ± 2.69	1.19 ± 2.75
	t	3.597	4.836	3.529	3.648	1.648	1.648
	P	< 0.001	< 0.001	0.001	< 0.001	< 0.001	< 0.001

### Asymmetry rate

After traction therapy, the asymmetry between the submaxillary suture minimum point (INM), piriformis lateral margin point (LPA) and pterygomaxillary suture point (PTM) was significantly smaller than that before traction therapy (P < 0.05), and the asymmetry rate was lower in the observation group than in the control group (P < 0.05), as shown in Table 3.

**Table 3:** Asymmetry rates (x̅±s, %)

	Control group		Observation group		t	P
Landmark point	T0	T1	T0	T1		
INM	2.18 ± 1.38	1.26 ± 0.99	2.19 ± 1.42	1.36 ± 1.64	2.824	0.006
LPA	7.86 ± 4.13	4.34 ± 2.73	7.49 ± 4.26	5.09 ± 3.48	5.996	<0.001
PTM	14.39 ± 9.79	5.81 ± 3.04	13.62 ± 9.06	8.34 ± 2.21	4.142	<0.001

## Discussion

Patients with unilateral cleft lip and palate disease differ in their jaw histomorphology distribution on the cleft side and those on the non-cleft side 5. At the same time, there are also some differences in the degree of muscle tension between the two sides, which in turn brings a significant inhibitory effect on the growth and development of maxillary tissue on the cleft side and gradually evolves into a midface depression deformity with typical characteristics and asymmetry, significantly improving the difficulty coefficient of clinical corrective treatment work 6. At present, the clinical treatment of unilateral cleft lip and palate severe midface skeletal hypoplasia in the disease is mainly treated with osteotomy traction osteogenesis, through the use of external traction device to promote the body's maxillary tissue suitable for forward movement, on this basis, the bone block movement mode is correspondingly improved and optimized, and finally achieve the purpose of improving the coordination and symmetry between the patient's facial tissues of clinical treatment. However, this treatment will cause significant traumatic effects on patients after surgical treatment, but also have a certain degree of interference effect on the potential of their body's bone growth and development.

Compared with the non-slit side, it can be seen that the pressure produced by the labiobuccal soft tissue and palatal scar tissue endured by the slit side with relatively small volume has a relatively greater effect, which in turn causes the development of the maxillary segment tissue on the slit side to be ideal, which evolves into a midface depression deformity disease with typical clinical symptoms over time and has asymmetric characteristics 7.

Pan et al. 8 showed through the study of the symmetrical relationship expansion system of the midface skeleton that the asymmetric deformity of the midface skeleton in unilateral cleft lip and palate mainly occurred in the young stage, while it was mainly distributed in the position near the cleft as well as the position of the zygomatic ridge. For this purpose, in this study, the lowest point of the maxillary suture (INM), the lateral margin of the piriformis (LPA), and the pterygomaxillary suture point (PTM) were considered as the study marker points, and the location of the marker points on each slit side was significantly posterior before traction treatment. However, after the completion of traction therapy, although the difference in the lateral margin of the piriformis foramen (LPA) was significantly reduced, the position of the slit side was still relatively posterior compared with the nonslit side. Comparative analysis of the asymmetry rate of the above marker points before and after traction shows that they are significantly reduced 9. Thus, the canine pillar bone bearing traction method of skull external fixator combined with nickel-titanium memory alloy spring can significantly promote the degree of asymmetric depression deformity in the midface of patients with cleft lip and palate disease to be significantly improved 10,11.

In addition, in order to better avoid the recurrence of the disease after surgery, this study uses the rich experience accumulated in the process of osteotomy traction treatment to carry out appropriate traction correction treatment for patients and corresponding orthodontic treatment according to the actual situation of patients and other related measures. Among them, the use of traction overcorrection measures for intervention, one can significantly improve the poor effect of tooth occlusal relationship establishment after the end of surgery, minimize the possibility of early disease recurrence, the other can also effectively promote the growth and development of mandibular tissue. In this study, scientific orthodontic treatment and facial arch traction maintenance treatment measures can adjust and optimize the adverse tooth occlusal relationship produced by patients during treatment, minimizing the negative effects produced by later mandibular tissue called ghrelin. In addition, the canine pillar bone bearing traction method of skull external fixator combined with nickel-titanium memory alloy spring is suitable for the clinical treatment of unilateral cleft lip and palate severe midface skeletal hypoplasia. It can use the elastic traction measures of facial arch to promote the maintenance of maxillary advancement and effectively inhibit the recurrence of the disease. After the treatment, the bone metabolism activity and bone remodeling activity in the inner suture area still have a high activity, and there is also a certain degree of remodeling ability and adaptability in the bone suture. This treatment method retains a certain growth potential, which can effectively promote the facial arch traction maintenance and sustainable forward movement can be effectively realized.

Distraction osteogenesis is a minim invasive and relatively low-risk treatment option associated with minimal scarring, compared to other bone lengthening and reconstruction procedures. The procedure allows for gradual, precise bone lengthening making it a safe and reliable procedure with a high success rate. The process allows for the patient to regain function of the treatment area faster than with more invasive procedures. Multiple bones can be treated at the same time with this method. But Recovery time can be lengthy, depending on the treatment area and amount of healing required. The process can be time-consuming and may require multiple doctor visits to monitor progress. The patient must be compliant with the post-treatment protocol in order for the treatment to be successful. There is a risk of developing complications due to tissue overstretching and misalignment.

## Conclusion

In summary, the canine pillar bone bearing traction method of skull external fixator combined with nickel-titanium memory alloy spring is used in the clinical treatment of unilateral cleft lip and palate with severe facial skeletal hypoplasia, providing a reasonable and scientific treatment for the early corrective treatment of this disease, and the clinical efficacy is more obvious.

## Data Availability

The datasets used and analysed during the current study are available from the corresponding author on reasonable request.
